# Preparation of a Sensor Based on Biomass Porous Carbon/Covalent-Organic Frame Composites for Pesticide Residues Detection

**DOI:** 10.3389/fchem.2020.00643

**Published:** 2020-08-28

**Authors:** Yali Liu, Mingyue Zhou, Chen Jin, Jinxiang Zeng, Chao Huang, Qiuye Song, Yonggui Song

**Affiliations:** ^1^Laboratory Animal Science and Technology Center, College of Science and Technology, Jiangxi University of Traditional Chinese Medicine, Nanchang, China; ^2^Pharmacy Department of Zhangjiagang, First People's Hospital, Suzhou, China

**Keywords:** sensor, AChE, integrated electrode, biomass carbon materials, detecting

## Abstract

In this work, a covalent-organic framework with high carbon and nitrogen content microstructures (named COF-LZU1), assisted by 3D nitrogen-containing kenaf stem composites (represented as COF-LZU1/3D-KSCs), was constructed. Moreover, it was utilized for immobilizing acetylcholinesterase (AChE) for identifying trichlorfon, a commonly applied organophosphorus (OP) pesticide. The development of COF-LZU1/3D-KSC was affirmed by SEM, PXRD, and EDXS. The findings confirmed that COF-LZU1 microstructures were uniformly developed on 3D-KSC holes using a one-step synthesis approach, which can substantially enhance the effective surface area. Also, the COF-LZU1/3D-KSC composite contains not only the nitrogen element in COF-LZU1 but also the nitrogen element in 3D-KSC, which will greatly improve the biocompatibility of the material. The AChE/COF-LZU1/3D-KSC integrated electrode was fabricated by directly fixing a large amount of AChE on the composite. At the same time, the integrated electrode had good detection efficiency for trichlorfon. Improved stabilization, a wide-linear-range (0.2–19 ng/mL), and a lower detection limit (0.067 ng/mL) have been displayed by the sensor. Therefore, this sensor can be used as an important platform for the on-site detection of OP residue.

## Introduction

Organophosphorus pesticides (OPs), such as Trichlorfon, have been thoroughly used in agriculture due to their powerful insecticidal ability (Ma et al., 2018). However, due to its inhibition of acetylcholinesterase (AChE), the key enzyme of nerve conduction (Baldissera et al., 2019), it also poses a major threat to overall health (Soreq and Seidman, 2001; Shi et al., 2016b). Consequently, quick and sensitive probes of OPs in food production have turned out to be of considerable importance. Conventional analytical techniques, like HPLC and gas chromatography, mostly combined with mass-selective detectors (Liu et al., 2017; Song et al., 2019a), are slow and costly. These approaches are still carried out in laboratories (Shi et al., 2016a; Liu et al., 2017; Su et al., 2018) butthey are not appropriate for quick field detection (Su et al., 2014; Song et al., 2019b). Therefore, the fabrication of rapid and sensitive OPs detection strategies with fewer limitations are increasingly desired by the food industry and for environmental monitoring.

An electrochemical AChE biosensor has the potential to replace traditional methods due to its higher sensitivity, fast response, and tiny volume (Zeng et al., 2019). According to the inhibition of OPS on AChE, even smaller concentrations of pesticide can be determined accurately. The sensitiveness and limit of detection of such biosensors is dependent on the amount of enzyme (Zhang et al., 2019), so enzyme immobilization on the electrode surface is a key step for biosensor activity.

In order to firmly immobilize the enzyme, many smart materials, such as carbon nanotubes (Sotiropoulou and Chaniotakis, 2005) and gold nanoparticles, were employed to fabricate an enzyme-entrapped matrix (Wang et al., 2003; Shi et al., 2019). The incorporation of enzymes in new nanomaterials effectively increased the stability, sensibility, and detection threshold of enzymatic biosensors. Nevertheless, several proteases will stack on the surface of these nanomaterials, which will affect the transmission of electrons and reduce the performance of sensors (Khalilzadeh et al., 2016). Therefore, it is very important to discover an electrode material that can modify a large number of proteases without the stacking effect while maintaining good biocompatibility. Carbon biomass materials have good electrical conductivity and biocompatibility and are very suitable for the preparation of electrode materials for enzyme biosensors (Song et al., 2015; Khalilzadeh et al., 2016; Su et al., 2020). However, due to the large pore size of the biomass carbon material, the transmission of electrons will be affected. Therefore, for bioelectrochemical enzyme sensors, it is crucial to modify micro-materials with good conductivity and biocompatibility in the holes.

In this study, metal-free frame microstructures utilizing a covalent organic framework (named COF-LZU1) through the (3D N-containing kenaf stem) composites were formed using a one-step method. The COF-LZU1s may spread over the pores of 3D-KSC, and also had good biocompatibility because they contain no metal elements and only carbon, nitrogen, and oxygen elements. The COF-LZU1sshowed pitted surfaces, which, when superimposed with the 3D porous structure of KSC, can be employed to entrap more AChE molecules. Furthermore, AChE molecules were added in the COF-LZU1s by using pits of COFs, that efficiently prevented the agglomeration of enzymes at the electrode surface. Moreover, COF-LZU1s material also has good conductivity (Liu et al., 2016; Song et al., 2016a), which can improve the proton transportability of the whole integrated electrode. Thus, the developed trichlorfon sensor based upon the AChE/COF-LZU1/3D-KSC composites showed a wide-range linearity, lower detection limitations, and good stability.

## Experimental Section

### Materials and Reagents

The kenaf stems (KS) were collected from the Futian farm in Ji'an, Jiangxi Province. Graphite powder (99.95% and 325 mesh) and paraffin were acquired from Aladdin. DMFc, 1,3,5-triformylbenzene, acetylthiocholine chloride (ATCl), 1,4-diaminobenzene, and Acetylcholinesterase (1,000 U/mg), were obtained from Sigma-Aldrich (USA). Trichlorfon was bought from Kanghe Yinong Biotechnology Co., Ltd. Other reagents utilized were of analytical grades and procured from Shanghai Guoyao Group Chemical Reagent (China). Distilled water (18.2 MΩ cm) was employed for making all the solutions and purged by nitrogen prior to experiments. PBS was freshly made using dihydrogen phosphate and sodium disodium hydrogen phosphate.

### Instruments

Cyclic voltammetry (CVS) and differential pulse voltammetry (DPVS) were carried out on the CHI660E electrochemical analyzer. A three-electrodes system with a platinum wire (auxiliary electrode), a saturated calomel electrode (SCE) (reference electrode), and AChE/COF -LZU1/3D-KSCE was adopted as a working electrode. CVs and DPVs were carried out in 10 mL (0.2 M PBS of pH 7.0) under 25°C. SEM was done employing an XL30 ESEM-FEG SEM using accelerating voltage (20 kV) provided with a Phoenix (EDXA). The PXRD data was gathered over a (D/Max 2,500 V/PC) diffractometer via Cu Kα radiation info (λ = 0.154056 nm, 40 kV, and 200 mA).

### Preparation of COF-LZU1/3D-KSCs Composite

The carbonization of dried KS synthesized the 3D-KSC in a high-heating furnace following protocol from our former project (Song et al., 2015; Khalilzadeh et al., 2016). The carbonization procedure was executed in a quartz reactor in an N_2_ environment on heating (5°C min^−1^) and annealing (2 h at 900°C). 3D-KSC was split up to a cylindrical shape, with the exterior diameter equivalent to the inner diameter of a used pipette tip. Therefore, the cylindrical 3D-KSC can be immobilized firmly in the already treated pipette tip. The prepared 3D-KSC was treated with dilute hydrochloric acid (2 M) for 24 h, and distilled water (24 h) to eliminate the inorganic contaminants, and afterwards was cleaned with ethanol and purified water successively, dried out normally, and placed in a beaker. Subsequently, 1,3,5-Triformylbenzene (0.30 mmol) and 1,4-dia-minobenzene (0.45 mmol) were measured and solubilized in 3 mL of 1,4-dioxane. After that 3D-KSCs were immersed in the solution, shifted in a glass vial (volume 20 mL), and then 0.6 mL of 3 mol L^−1^ dilute acetic acid was added to the mixture. The glass vial was flash-frozen in liquified nitrogen, subjected to a 19 mbar of internal pressure and flame-sealed, decreasing 10 cm in length. After attaining 25°C, the suspension was kept inside an oven uninterrupted for 3 days at 120°C, resulting in a yellow solid forming across the tube. The modified 3D-KSCs which were obtained after centrifugation were washed with N, N-dimethylformamide (3 × 10 mL) and tetrahydrofuran (3 × 10 mL), and then dried at 80°C in a vacuum for (2 h to produce COF-LZU1, a yellow-colored powder (90% yield), and produce the COF-LZU1-modified 3D-KSCs (COF-LZU1/3D-KSCs). Following our previous work,^14,15^ 3D-KSC were developed by carbonizing dried KS in a higher heating system. The procedure is explained as follows: in a tubular quartz reactor, carbonization is conducted at a rate of 5°C min^−1^ in an N_2_ atmosphere, and annealing is carried out at 900°C for 2 h. The 3D-KSC is made into a cylinder such that the outer diameter corresponds to the inner diameter of a processed pipette tip so that the cylindrical 3d-ksc can be firmly fixed on the treated pipette tip. After treatment, the 3D-KSCs were treated with diluted hydrochloric acid (2 M) and distilled water for 24 h to eliminate inorganic impurities, and after that were washed alternately with ethanol and ultrapure water, then dried and placed in a beaker. Then, 1,3,5-trimethyl benzene (0.30 mmol) and 1,4-diaminobenzene (0.45 mmol) were put in vials and dissolved within 3 mL 1,4-dioxane. Then 3D-KSCs was immersed in the solution, the mixture transferred to a glass ampoule (vol 20 ml), and 0.6 ml of 3.0 mol L^−1^ water acetic acid was added to the mixture. The glass ampoules were quickly frozen in a liquified nitrogen bath, vacuumed to 19 mbar interior pressure, and flame-sealed to reduce the entire length by 10 cm. After the suspension was heated to room temperature, it was put in an oven at 120°C for 3 d. The yellow solid was generated along the test tube. The modified 3D-KSCs were separated by centrifugation, and afterwards washed with N, N-dimethylformamide (3 × 10 mL) and tetrahydrofuran (3 × 10 mL). After vacuum drying at (80°C) for 12 h, the yellow powder COF -LZU1 (90% yield) was obtained. The modified 3D-KSCs of COF -LZU1 (COF -LZU1/3D-KSC s) were obtained.

### Preparation of Integrated AChE/COF-LZU1/3D-KSC Electrodes

**Scheme 1:**
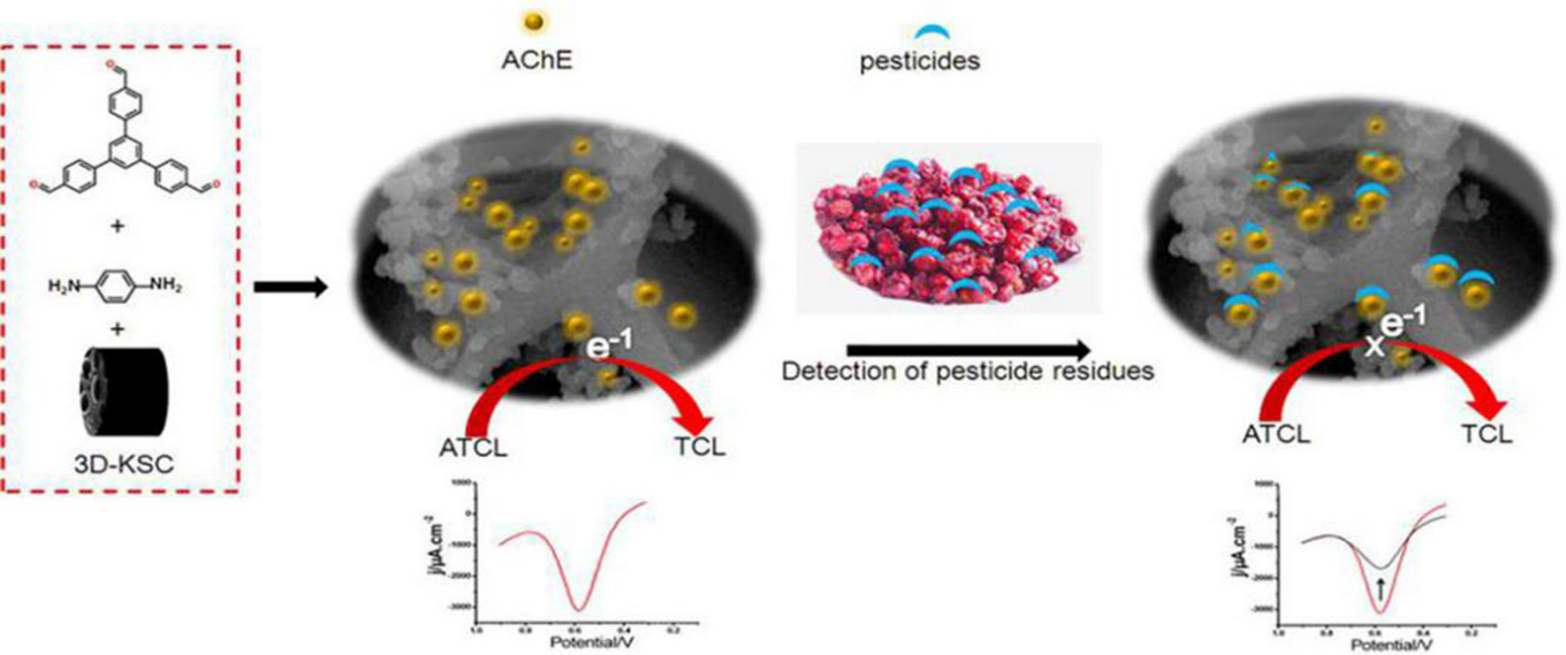
Schematic of the AChE/COF-LZU1/3D-KSC electrochemical pesticides biosensor.

The COF-LZU1/3D-KSC were incorporated within the processed pippete tip. After that, 0.25 g liquid paraffin was mixed with 1 g powder of graphite and homogenized for 20 min in the agate mortar. Then, the mixture was packed inside the upper portion of the pipette tip to touch the base of COF-LZU1/3D-KSC. Then, a copper wire was inserted into the end of the pipette tip, and connected with the COF-LZU1/3D-KSC at the tip through graphite paste. After the paste was naturally dried at room temperature, as depicted in Figure S1, the copper wire was further fixed with a sealing film or epicote. The AChE/COF-LZU1/3D-KSC electrode was fabricated by dropping a 5 μL AChE solution with various concentrations upon the electrode surface, followed by dessication. The entire preparation process was illustrated by Figure S1, including Scheme 1. Lastly, the modified electrode was washed with purified water to eliminate loosely bounded materials and kept at 4°C, intended for further usage. The obtained AChE/COF-LZU1/3D-KSC electrode was denoted as AChE/COF-LZU1/3D-KSCE.

### Inhibition Measurement of AChE Biosensor

The trichlorfon assay process was illustrated in detail in Scheme 1. Regarding inhibitory tests, the first (DPV) signal was (*I*_P,control_) recorded in 0.1 M PBS of pH 7 alongwith 1 mM ATCl. After that, the electrode was cleaned with distilled water, then placed inside an aqueous solution having a preferred concentration of trichlorfon for about 10 min. Afterwards, residual signal (*I*_P,exp_) was also observed in a similar state. The rate of trichlorfon inhibition was computed below:

In Scheme 1, the analysis process of trichlorfon was described in detail. For the inhibition test, the original (DPV) signal (*I*_P,control_) was determined in 0.1 M PBS (pH 7) and 1 mM ATCI. The electrodes were then cleaned by water and stored within an aqueous solution having the required amount of trichlorfon for 10 min. Following an incubation period, residual signals (*I*_P,exp_) were recorded under the same conditions. The inhibition rate of trichlorfon was estimated as below:

(2.1) $$\begin{array}{l} \text{Inhibition }\left(\text{\%}\right)=100\%\;\times \frac{I_{\text{P},\text{control}}-\;I_{\text{P},\text{exp}}}{I_{\text{P},\text{control}}} \end{array}$$

## Results and Discussion

### Characterization of AChE/COF-LZU1/3D-KSC Electrodes

**Figure 1:**
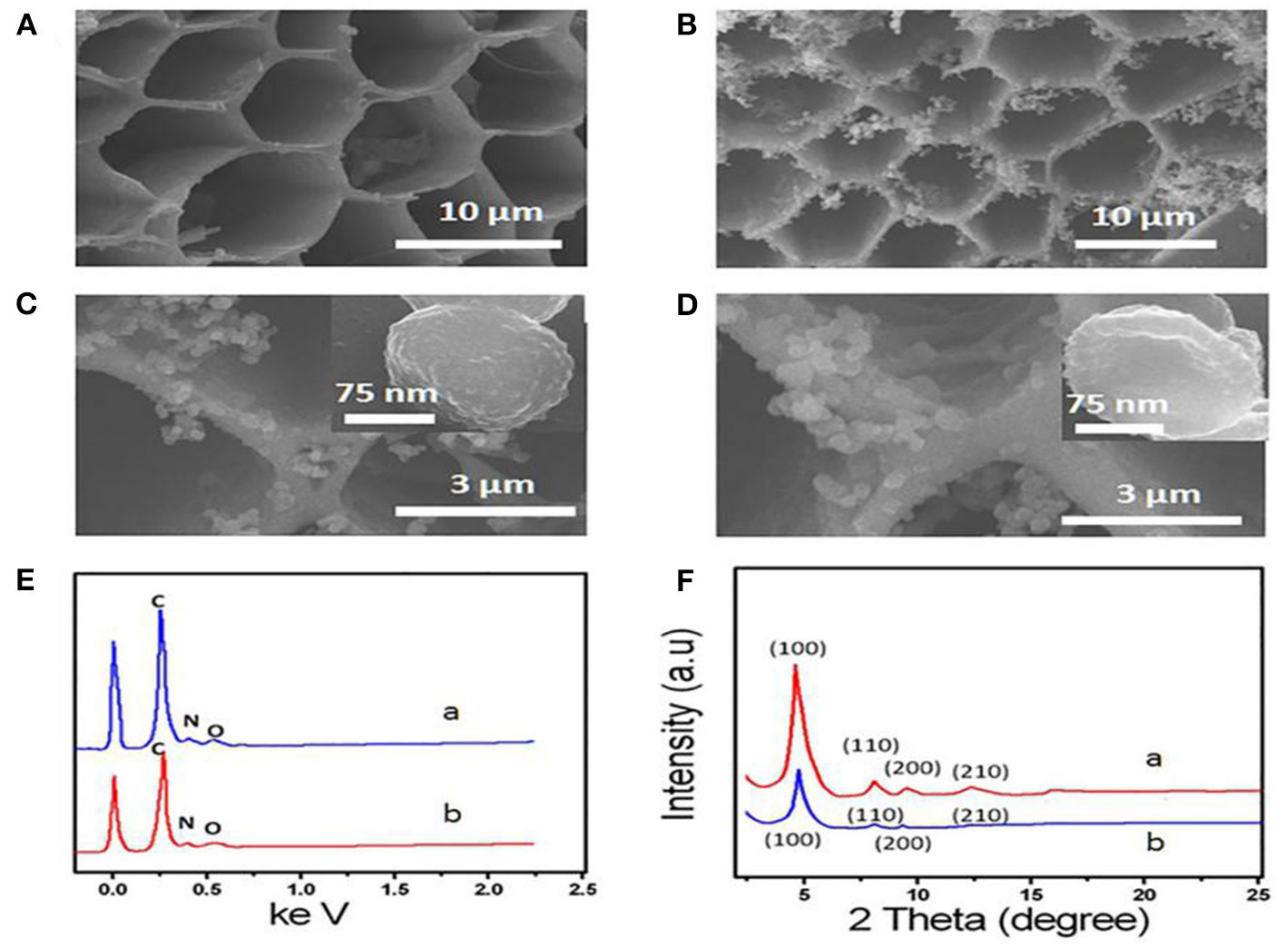
**(A)** SEM image of 3D-KSC and **(B,C)** SEM images of COF-LZU1/3D-KSC. **(D)** SEM images of AChE/ COF-LZU1/3D-KSC. **(E)** EDS curve of COF-LZU1(curve a) and COF-LZU1/3D-KSC (curve b). **(F)** XRD pattern of COF-LZU1(curve a) and COF-LZU1/3D-KSC (curve b).

Figures 1A–C shows the SEM images of 3D-KSC and COF-LZU1/3D-KSC composites. 3D-KSC have a 3D macroporous inner structure (Figure 1A) (Song et al., 2015; Shan et al., 2019). After the growth of COF-LZU1, the procured electrode surface was adequately coated with the COF-LZU1 microstructures (Figures 1B,C). As depicted in Figure 1C, the spherical COF-LZU1 microstructures size is about 150 nm (inset of Figure 1C). The high magnification image in Figure 1C shows a special bumpy morphology, which significantly enhances the surface of electrode and the mass transfer. The EDX spectrums of COF-LZU1 and COF-LZU1/3D-KSC indicate the higher pureness of the composite, containing only O, N, and C (Figure 1D). Simultaneously, it can be seen that not only KSC but also COF-LZU1 contain nitrogen, which may greatly increase the biocompatibility of the composites. Figure 1F displays the XRD pattern of COF-LZU1 and also COF-LZU1/3D-KSC, which shows a microcrystalline solid with a long-range structure. Moreover, diffraction peaks around 4.9, 8.0, 9.4, and 12.1 according to 100, 110, 200, and 210 crystal planes, in accordance with the reported literature (Song et al., 2016b). Also, the XRD diffraction pattern of COF-LZU1/3D-KSC coincides with that of COF-LZU1, which indicates that COF-LZU1/3D-KSC has the same crystal structure as that of a single COF-LZU1 material. When AChE molecules were collected on the COF-LZU1/3D-KSC electrode, the interior wall of pores in the COF-LZU1/3D-KSC electrode became rough and irregular, comprising of an opaque film of a fuzzy-like material which might have resulted from the adsorption of AChE molecules at the inner side of pores (Figure 1E). Furthermore, the structure of AChE molecules could be damaged whenever an electron beam pierced the protein, and consequently, the pore surfaces of the COF-LZU1/3D-KSC composite became fuzzy. The findings clearly established the effective immobilization of AChE molecules on the COF-LZU1/3D-KSC electrode.

### Electrochemical Behaviors of AChE/COF-LZU1/3D-KSC Electrodes

**Figure 2:**
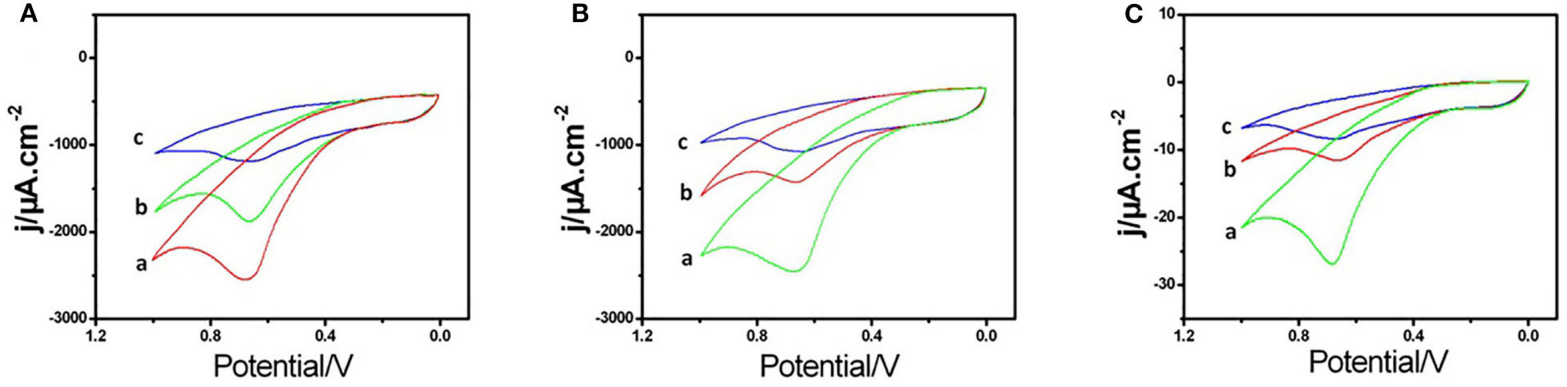
CVs of the **(A)** AChE/COF-LZU1/3D-KSC, **(B)** AChE/3D-KSC, and **(C)** AChE/GCE in PBS (pH 7.0) containing (1.0 mM ATCl) following 10 min incubation in 0.0 (curve a), 9.0 (curve b), and 18 ng/mL (curve c) trichlorfon solution.

For exploring the electrochemical characteristics of the AChE/COF-LZU1/3D-KSCE, the CVs of multiple electrodes, particularly AChE/COF-LZU1/3D-KSCE, AChE/3D-KSCE and AChE/glass carbon electrode (AChE/GCE), were investigated (Figures 2A–C). Figure 2A, curve a, showed the CVs of AChE/COF-LZU1/3D-KSCE in PBS (pH 7) having 1 mM ATCl. The CV of the AChE/3D-EUSE presented an irreversible oxidation peak on 0.68 V (curve a), resulting from thiocholine oxidation, the hydrolyzed material of ATCl, through enzyme catalysis. Contrarily, the maximal current with AChE/glass carbon electrode (AChE/GCE) (Figure 2C, curve a) was much lower. The increased response might be due to the stack effect of the COF-LZU1/3D-KSC composite, possessing a higher surface area that can immobilize additional enzymes. Meanwhile, COF-LZU1/3D-KSC also has the benefit of fast electron transfer owing to its 3D-porous composite structure. Additionally, the good biocompatibility of COF-LZU1/3D-KSC could well-preserve the highest bioaction by immobilized enzymes. Following 10 min placing in 9.0 ng/mL and 18 ng/mL trichlorfon solution, the anodic peak currents (curves b and c, Figure 2A) were significantly reduced compared to the control (curve a Figure 2A), and the reduction in peak current improved with the rising concentration of trichlorfon. It was because trichlorfon, an OP compound, displayed acute toxicity and produced an irreversible inhibitory response upon AChE, which therefore decreased enzymatic action to its substrate. However, the anodic peak currents of AChE/3D-KSCE (curves b and c, Figure 2B) and AChE/GCE (curves b and c, Figure 2C) decreased irregularly. This may be due to the decrease of enzyme modification, resulting in the narrowing of the detection range (Figure 2B) and could also be related to the fact that the glassy surface of the carbon electrode is smooth and the AChE can not be immobilized for a long time (Figure 2C). The trichlorfon concentration can be determined by changes in the voltammetric signal of the AChE/COF-LZU1/3D-KSCE. The principle of detection was pictorially illustrated by Scheme 1.

### Influence of pH Value, ATCl, and AChE Concentration

**Figure 3:**
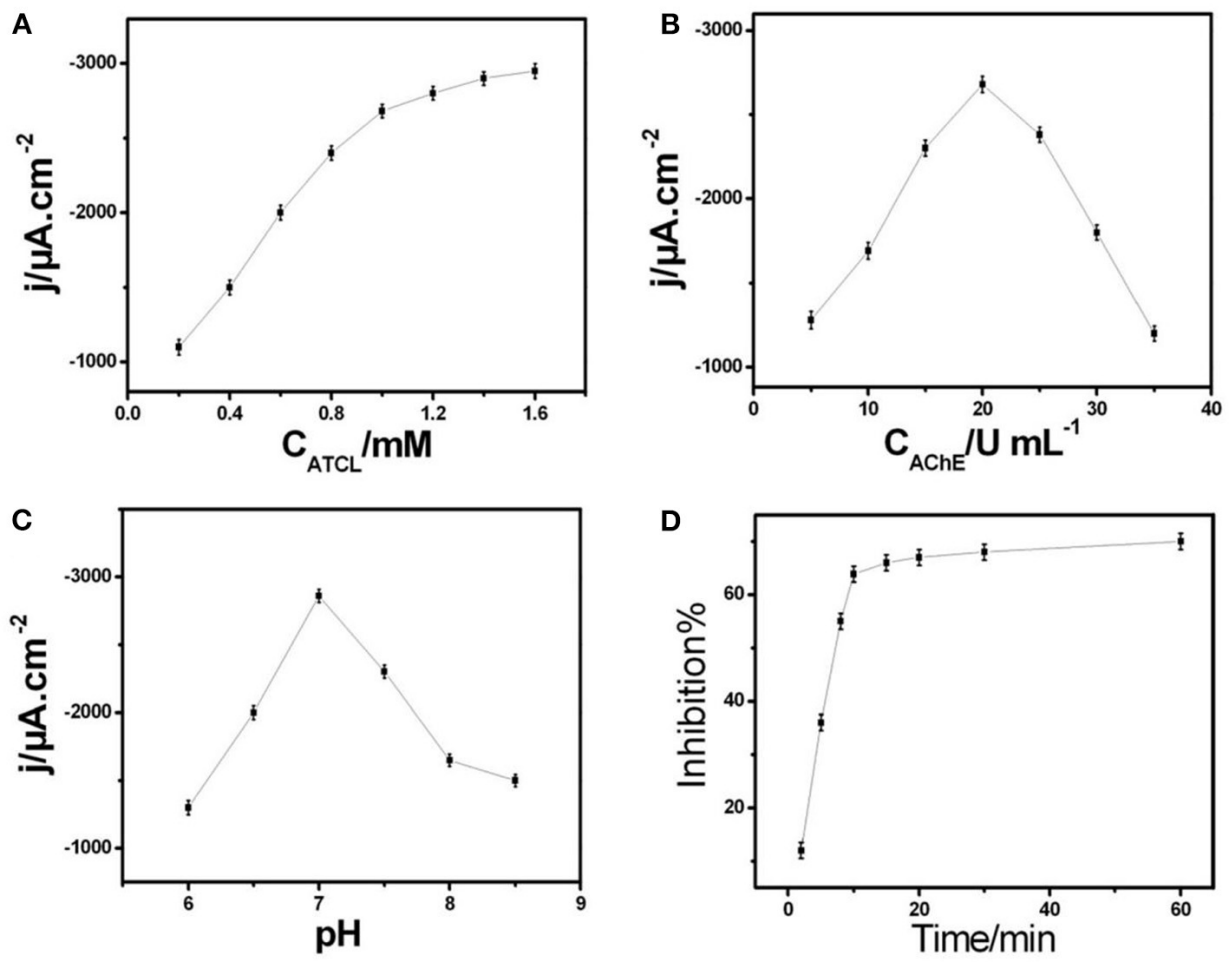
**(A)** Correlation among current response to ATCl concentration in 0.1 M PBS of pH 7. **(B)** Plot of amperometric response vs. AChE concentration of AChE/COF-LZU1/3D-KSCE in 0.1 M PBS of pH 7 with 1 mM ATCl. **(C)** Effect of pH on current response of AChE/COF-LZU1/3D-KSCE to 1 mM ATCl. **(D)** Effect of inhibition time on inhibition (percentage) of AChE/COF-LZU1/3D-KSCE in 0.1 M PBS of pH 7 with 1 mM ATCl; inhibition of trichlorfon was 19 ng mL^−1^.

Figure 3A showed the ampere sensitivity of AChE/COF-LZU1/3D-KSCE after adding ATCl. The usual biosensor current-time (response curve) was achieved when adding a substrate continuously in the stirred tank. By increasing ATCl concentration, the current response improved and tended to be stable at 1.0 mM. It might be due to the increase of ATCl concentration which leads to the active-sites' saturation of the enzyme by ATCl, thus reducing the binding sites of new molecules. The growth rate of peak current then shows a downward trend. Therefore, in the next pesticide analysis experiment, 1.0 mM ATCl was selected as the constant concentration.

The immobilization of AChE upon the surface electrode is another essential factor affecting the biosensor efficiency. Figure 3B showed the relationship between AChE concentration and the biosensor (amperometric response). With an increasing AChE concentration, a gradual increase occured in peak current and attained the highest value at around 20 U mL^−1^. After this point, further addition of AChe will slowly weaken the current response. Its behavior may be ascribed to the presence of lesser AChE amounts, which is not enough to catalyze substrate oxidation, while too thick an AChE-modified layer may hinder mass and electron transfer, thus reducing the catalytic current. Therefore, this turning point may be due to the inhibition of COF-LZU1/3D-KSC to generate thiocholine and electron transfer by a large number of AChE. Therefore, in the next experiment, 20 U mL^−1^ AChE solution was used to build AChE/ COF-LZU1/3D-KSCE.

For electrochemical biosensors, pH value is the key factor that affects their stability and sensitiveness. Therefore, the influence of pH value was also studied. As shown in Figure 3C, at pH = 7, the maximum ampere response of AChE/COF-LZU1/3D-KSCE at 1 mM ATCl was obtained, which is consistent with most reported AChE biosensors (Ding et al., 2011; Su et al., 2016). Among the most influential parameters in pesticide assay is the culture time of inhibition. While increasing the incubation time period, there is also an increase in the rate of inhibition. Whereas, the required time of inhibition has been determined on various time-intervals varying from 02 to 60 min (Figure 3D). By prolonging the incubation timeframe, the rate of inhibition elevated to its highest value after incubating with trichlorfon 19 ng/mL for 10 min. Therefore, 10 min is used for the test.

### Voltammetric Detection of Trichlorfon

**Figure 4:**
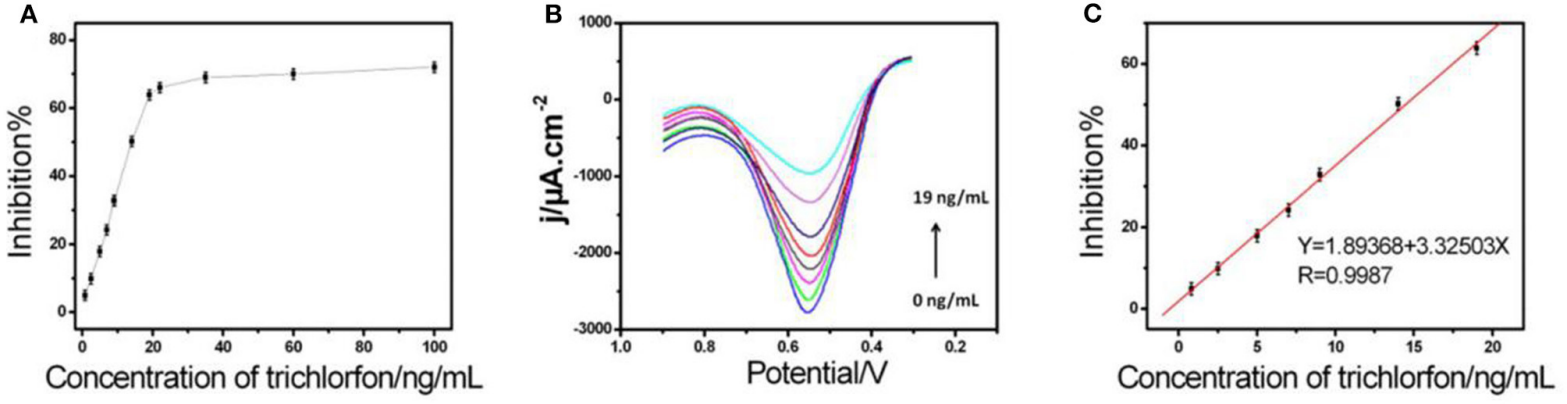
**(A)** The biosensor inhibition curve with varying concentrations of trichlorfon (inhibitions corresponded to trichlorfon concentrations of 0.8, 2.5, 5, 7, 9, 14, 19, 22, 35, 60, and 100 ng mL^−1^, respectively), in 0.1 M PBS with pH 7 consisting of 1 mM ATCl. **(B)** DPVs and **(C)** standard curve for trichlorfon assessment in 0.1 M PBS of pH 7 including 1 mM ATCl.

In an optimized state, the inhibition stayed proportionate to varying concentrations of trichlorfon with 0.20–19 ng/mL (Figure 4), having a limit of detection (0.067 ng/mL). The effectiveness of AChE/COF-LZU1/3D-KSCE compared to other stated AChE biosensors is listed in Table 1, which indicated that the current AChE/COF-LZU1/3D-KSCE exhibited an equivalent or lower detection limit, demonstrating that COF-LZU1/3D-KSCE had multifunctions in enzyme immobilization. The high carbon content and nitrogen doted characteristics may sustain the enzymatic activity; moreover, the high specific surface-area with excellent electrical conducting potential of the composite would help a lot improvementn improving the sensitivity.

**Table 1:** Comparative evaluation of various AChE biosensors' efficiency used for pesticide detection.

**Process**	**Detected pesticide**	**Linear range**	**Limit of detection**	**Reference**
Nafion/AChE/Chit-PB-MWNTs-HGNs/Au	Carbofuran	1.11–17.70 ng/mL	0.55	Kandimalla and Ju, 2006
AuNPs-MWCNTs-chitosan	Monocrotophos	0.1–10 μM	10 nM	Zhai et al., 2013
AChE-(xGnPs)-chitosan	Parathion	0.005–0.039 μM	0.158 nM	Norouzi et al., 2010
NF/AChE-CS/SnO_2_NPs-CGR-NF/GCE	Carbofuran	2.21 × 10^−4^-2.21 × 10^−2^ ng/mL 2.21 × 10^−2^−2.21 ng/mL	1.11 × 10^−4^ ng/mL	Ion et al., 2010
AChE/e-GON-MWCNTs/GCE	Carbofuran Paraoxon	0.03–0.81 ng/m 0.05–1, 1−104 ng/mL	0.015 ng/mL 0.025 ng/mL	Zhou et al., 2013
AChE/Au-MWNTs/GCE	Paraoxon	0.028–1.927 ng/mL	0.028 ng/mL	Li et al., 2017
PPy-AChE-Geltn-Glut/Pt	Carbofuran Paraoxon	0.025–2, 5–60 ng/mL 0.1–12.5, 12.5–150 ng/mL	0.12 ng/mL 1.1 ng/mL	Jha and Ramaprabhu, 2010
AChE/SWCNT-Co phtalocyanine/GCE	Paraoxon	5–50 ng/mL	3 ng/mL	Dutta and Puzari, 2014
AChE/CNT-NH_2_/GCE	Paraoxon	0.055–0.275 ng/mL, 0.275–8.257 ng/mL	0.022 ng/mL	Ivanov et al., 2011
AChE/ZnO-MWCNTs-sG/GCE	Paraoxon	0.275–7.156 ng/mL	2.752 × 10^−4^ ng/mL	Yu et al., 2015
AChE/Fe_3_O_4_-CH/GCE	Carbofuran	1.11–19.91 ng/mL	0.80 ng/mL	Nayak et al., 2013
AChE/PAMAMb-Au/CNTs/GCE	Carbofuran	1.06–19.91 ng/mL	0.89 ng/mL	Jeyapragasam and Saraswathi, 2014
AChE/COF-LZU1/3D-KSCE	Trichlorfon	0.2–19 ng/mL	0.067 ng/mL	Current work

### Precision, Stability, and Selectivity of Biosensor

After trichlorfon (10 ng/mL) solution was added after around 10 min, the inter-assay precision of 1.0 mM ATCl was established on five distinct electrodes; the inter-assay precision was 3.9%, which proved that the precision and repeatability were good. The interference of several electro-active phenol derivatives (like nitrophenol, catechol, and hydroquinone) and the detection ofinorganic substances containing oxygen (${\text{SO}}_{4}^{2-}$, ${\text{NO}}_{3}^{-}$, sodium citrate) was also studied. As shown in Figure S2, when adding 2 times of nitrophenol, hydroquinone, catechol, ${\text{SO}}_{4}^{2-}$, ${\text{NO}}_{3}^{-}$, and Na_3_C_6_H_5_O_7_ in determining trichlorfon (19 ng/mL), the inhibition behavior did not change significantly. The good selectivity of the electrode is confirmed and can be utilized for determining actual amounts of trichlorfon in samples. The enzymatic electrode is placed in 4°C in a dry environment unless used. During the first 5 d of storage, the reaction of ATCl did not decrease significantly. After 30 d of storage, the current response of the sensor was still maintained (94%) at the primary response (Figure S3).

### Reactivity and Real Sample Analysis

Activation of AChE is a key additional component affecting the effectiveness of biosensors. Irreversible inhibiton of AChE by OPs could be fully activated by the use of nucleophilic agents like praldoximin chloride (PAM-Cl), while, 5 mM PAM-Cl PBS concentration was applied for activation. Then, the biosensor was dipped in the PAM-CL solution to inhibit trichlorfon. After 10 min of regeneration, AChE activity recovered completely. By reactivating the procedure, the biosensor can be reused up to five times with good constancy. The biosensor practicability was further proven by the addition of different amounts of trichlorfon to the Schisandra chinensis samples for the recovery test. Table S1 provides an overview of the results. The recovery was 96.1–105%. The results show that the method has high accuracy, high precision, and good reproducibility. It can be employed for the direct detection of associated samples.

## Conclusion

During the present project, a stable and highly sensitive biosensor was fabricated using an AChE-modified COF-LZU1/3D-KSC composite, which makes it possible to detect even trace amounts (0.067 ng/mL) of an organophosphorus compound trichlorfon. The use of COF-LZU1/3D-KSC has significantly enhanced the biosensor efficiency in three ways: (1) COF-LZU1s and porous 3D-KSC provides a synergestic response due to the fully bumpy and hollow surface area that can firmly immobilize additional enzymes; (2) COF-LZU1s significantly improves electrical signaling due to fast electron transfer; and (3) Derived nitrogen elements from COF-LZU1 and 3D-KSC show that the higher bioactivities of the immobilized enzymes are also maintained. Due to these factors, the developed biosensor exhibited tremendously high sensitivity and lower-detection limits, and thus is more reliable to detect trace residues of OP pesticide compared to other AChE biosensors.

## Data Availability Statement

The original contributions presented in the study are included in the article/Supplementary Material, further inquiries can be directed to the corresponding author/s.

## Author Contributions

YS conceived and designed the project. YL and MZ analysised experimental date and drafted the manuscript. CJ, JZ, and CH performed research. QS contributed methods and resources.

## Conflict of Interest

The authors declare that the research was conducted in the absence of any commercial or financial relationships that could be construed as a potential conflict of interest.
