# Supplementary figures and images for: Preparation of a Sensor Based on Biomass Porous Carbon/Covalent-Organic Frame Composites for Pesticide Residues Detection
Source: Front Chem. 2020 Aug 28;8:643. doi: 10.3389/fchem.2020.00643 (PMC7485226; doi:10.3389/fchem.2020.00643)

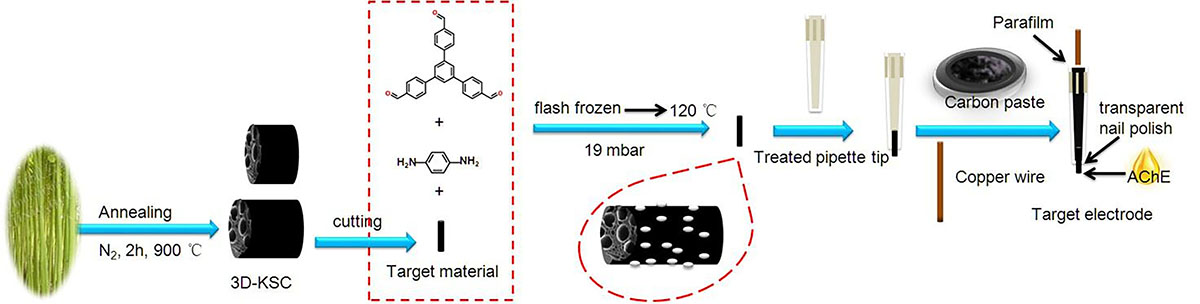

Supplement: Supplementary file 1 [file Image_1.JPEG]

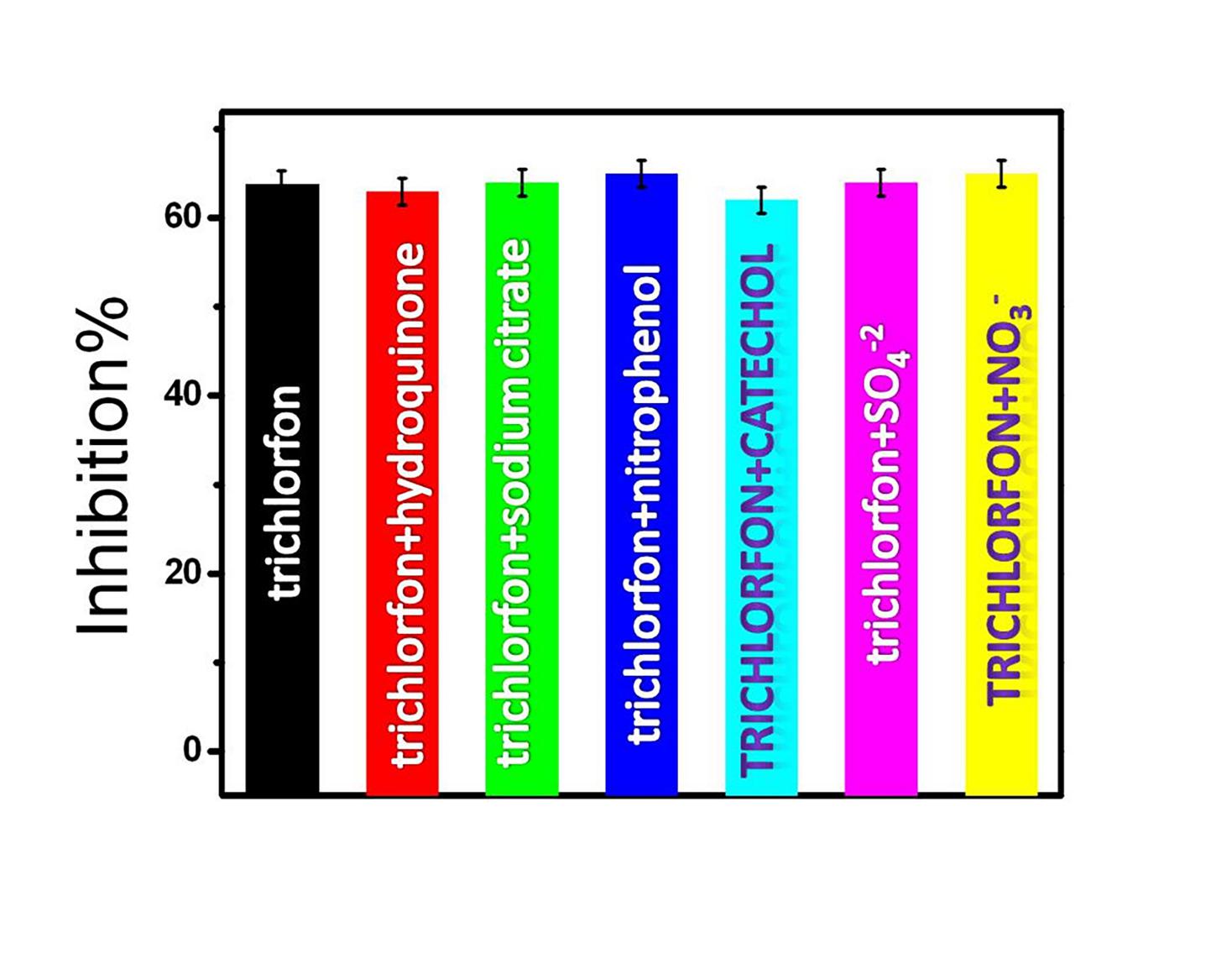

Supplement: Supplementary file 2 [file Image_2.JPEG]

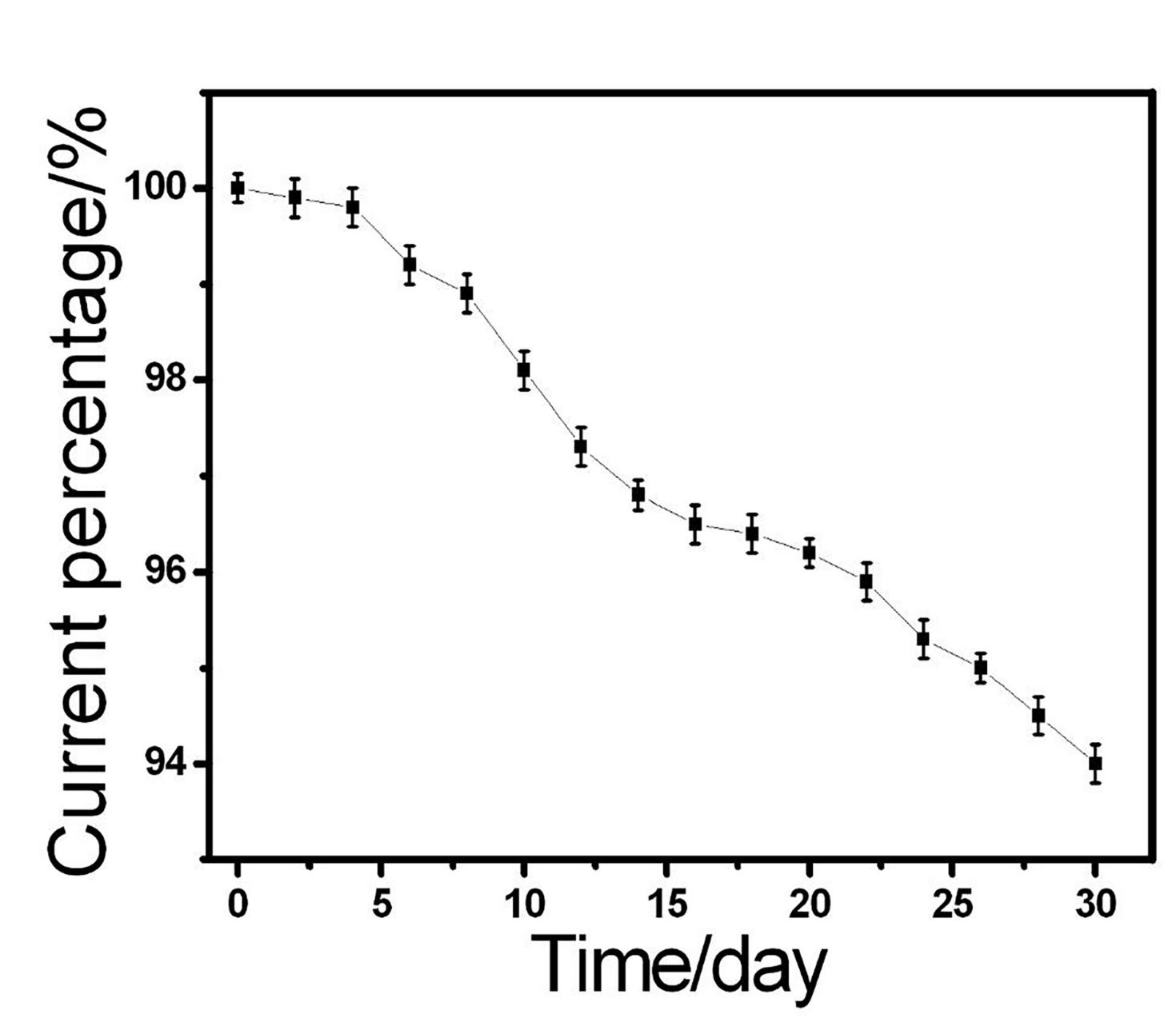

Supplement: Supplementary file 3 [file Image_3.JPEG]
