# Supplementary material for: Preparation of a Sensor Based on Biomass Porous Carbon/Covalent-Organic Frame Composites for Pesticide Residues Detection
Source: Front Chem. 2020 Aug 28;8:643. doi: 10.3389/fchem.2020.00643 (PMC7485226; doi:10.3389/fchem.2020.00643)
Supplement: Supplementary file 4 [file Table_1.DOCX]

**Supplementary information**

**Table S1.** Recovery studies of trichlorfon in schisandra chinensis samples. (Each result was estimated by six determinations)

| Sample | Taken  (ng mL^-1^) | Found  (ng mL^-1^) | Recovery  (%) | RSD  (%) |
| --- | --- | --- | --- | --- |
| 1 | 0.800 | 0.769 | 96.1 | 3.4 |
| 2 | 1.50 | 1.56 | 104 | 3.9 |
| 3 | 3.00 | 2.89 | 96.3 | 3.4 |
| 4 | 6.00 | 5.91 | 98.5 | 3.7 |
| 5 | 15.0 | 15.7 | 105 | 3.6 |
